# The Intestinal Cues Promoting Serotonin Release From the Human Gut

**DOI:** 10.1016/j.jcmgh.2025.101664

**Published:** 2025-10-18

**Authors:** Sara C. Di Rienzi

**Affiliations:** Center for Advanced Biotechnology and Medicine, Rutgers University, Piscataway, New Jersey; Department of Neuroscience and Cell Biology, Rutgers University, Piscataway, New Jersey; Rutgers Cancer Institute, Rutgers University, Piscataway, New Jersey

The intestine is the largest endocrine organ in the body and the primary source of the neurotransmitter and hormone serotonin. Intestinally derived serotonin regulates diverse functions, including motility, fluid secretion, and sensation,^1^ as well as food intake,^2^ and it also influences depression- and anxiety-related behaviors.^1^^,^^3^ Serotonin release in the gut is known to be triggered by microbial metabolites, mechanical stimuli, and other chemical signals within the intestinal ecosystem.^4^ Nutrients, in particular, are hypothesized to be key regulators of serotonin secretion. In a previous issue of *Cellular and Molecular Gastroenterology and Hepatology*, Alcaino and colleagues, in their article entitled "Mechanisms of Activation and Serotonin Release From Human Enterochromaffin Cells*,"*^5^ use human intestinal enteroids to characterize a wide range of dietary and intestinal metabolites that stimulate serotonin release. They further identify numerous receptors expressed on these cells and characterize their electrophysiological properties. This work provides new mechanistic insights into serotonin regulation in the human gut, with important implications for disorders affecting both the intestine and brain, including inflammatory bowel disease, irritable bowel syndrome, anxiety, and depression.

Most serotonin in the gut is produced by specialized enteroendocrine cells known as enterochromaffin cells, which are distributed throughout the intestinal tract. Previous work in mice characterized both the physiology and receptors of enterochromaffin cells,^4^^,^^6^ suggesting that their stimuli might vary by intestinal region. For example, enterochromaffin cells in the upper small intestine were found to express receptors for mechanical sensation, toxins, and nutrients, whereas those in the colon were predominantly found to express receptors for microbial metabolites.^6^ However, whether these findings extend to humans, and whether such predicted stimuli can activate human enterochromaffin cells, was not determined.

In their recent study, Alcaino et al^5^ addressed key gaps in enterochromaffin cell physiology by using human duodenal enteroids in which serotonin-producing cells were fluorescently labeled. Using flow cytometry to isolate these cells, the authors combined RNA sequencing and peptidomic analyses to identify peptide hormones produced in and receptors expressed in human enterochromaffin cells.

From these data, Alcaino et al next predicted and tested stimuli of human enterochromaffin cells. Highlighting the rigor of their research effort, they evaluated stimuli based on the 3 assays: promotion of (1) intracellular Ca^2+^, (2) cAMP, and (3) serotonin secretion. Their results revealed that the adrenergic receptor ADRB1 agonist isoproterenol promoted serotonin secretion, whereas the adrenergic receptor ADRA2 agonist clonidine did not, indicating a functional difference between mouse and human enterochromaffin cells. They next identified human intestinal hormones, bile acid receptor agonists, and specific amino acids that stimulate enterochromaffin cells. Notably, the satiety hormones CCK and GIP and the aromatic amino acid tryptophan stimulated serotonin release, whereas the intestinal hormones PYY and SST inhibited enterochromaffin activity.

Finally, Alcaino et al performed the first electrophysiological assessment of human enterochromaffin cells. Using perforated- and whole-cell voltage patch-clamp recordings, they found that these cells generate action potentials and depolarize during vesicle exocytosis, similar to other human enteroendocrine cells. They further demonstrated that chemical stimulation promotes vesicle exocytosis, consistent with their function as secretory cells.

Altogether, this study represents the first genomic and functional characterization of human enterochromaffin cells. It validated several genomic predictions of enterochromaffin cell function, including responsiveness to specific amino acids, bile acid receptor agonists, chemical irritants, and microbial short-chain fatty acids, and confirmed serotonin secretion in response to these stimuli. It also identified key differences between human and mouse enterochromaffin cells, including the aforementioned difference in adrenergic receptor function and in the repertoire of co-produced peptide hormones.

Future work can extend this research in several directions. First, the methods established here can be applied to direct therapeutic strategies for stimulating serotonin. Second, it remains unknown how enterochromaffin cells respond when multiple receptors are activated simultaneously. Third, whether human enterochromaffin cells functionally vary along the length of the gastrointestinal tract, as observed in mice, has yet to be determined. Fourth, the extent to which disease states alter enterochromaffin function remains an open question. Continued investigation of these cells will advance our understanding of gut–brain axis disorders and promote positive health outcomes.
